# Discovery of a hyperalkaline liquid condensed phase: significance toward applications in carbon dioxide sequestration

**DOI:** 10.3389/fbioe.2024.1382071

**Published:** 2024-04-30

**Authors:** Mark A. Bewernitz, Jacob Schneider, Christopher L. Camiré, Seung-Hee Kang, William L. Bourcier, Richard Wade, Brent R. Constantz

**Affiliations:** ^1^ Physical Science Department, College of Arts and Sciences, Embry-Riddle Aeronautical University, Daytona Beach, FL, United States; ^2^ Blue Planet, Ltd., Los Gatos, CA, United States

**Keywords:** carbon sequestration, liquid condensed phase, built environment, biomineralization, carbon star

## Abstract

Bicarbonate ion-containing solutions such as seawater, natural brines, bovine serum and other mineralizing fluids have been found to contain hyperalkaline droplets of a separate, liquid condensed phase (LCP), that have higher concentrations of bicarbonate ion (HCO_3_^−^) relative to the bulk solution in which they reside. The existence and unique composition of the LCP droplets have been characterized by nanoparticle tracking analysis, nuclear magnetic resonance spectroscopy, fourier transform infrared spectroscopy, dissolved inorganic carbon analysis and refractive index measurements. Carbon dioxide can be brought into solution through an aqueous reaction to form LCP droplets that can then be separated by established industrial membrane processes as a means of concentrating HCO_3_^−^. Reaction of calcium with the LCP droplets results in calcium carbonate precipitation and mineral formation. The LCP phenomenon may bear on native mineralization reactions and has the potential to change fundamental approaches to carbon capture, sequestration and utilization.

## Introduction

The partial pressure of CO_2_ (*P*_CO2_) in Earth’s atmosphere has varied considerably over Phanerozoic time. Oceanic bicarbonate ion (HCO_3_^−^) concentration and alkalinity of the ocean would have followed the changing *P*_CO2_. It is currently assumed that carbon in the oceans is in equilibrium with Earth’s atmosphere and that it comprises a single phase. By knowing any two of the variables *P*_CO2_, pH, dissolved inorganic carbon (DIC) and alkalinity, for a given temperature, salinity and pressure, all the other parameters can be calculated. Recent studies of calcium carbonate (CaCO_3_) solubility and formation in ocean water with changing *P*_CO2_, provide evidence for the effects of prenucleation clusters and phase separations of calcium (Ca^2+^) and carbonate ions (CO_3_^2−^) (Gebauer and Coelfen, 2011) that affect carbonate chemistry.

**FIGURE 1:**
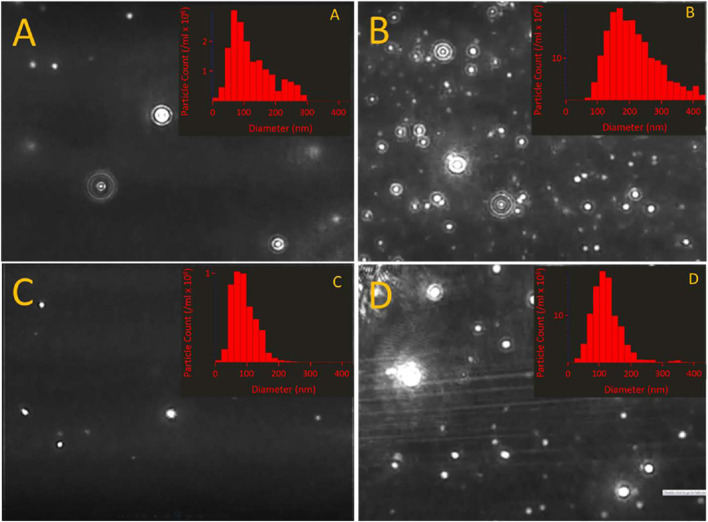
Size distribution of LCP droplets in various solutions at 25°C, as determined by nanoparticle tracking analysis (NTA). NTA is preferred over dynamic light scattering in characterizing the particle size distribution of LCP due to its sensitivity to dilute concentrations and its spatial resolution (Filipe et al., 2010), and has proven successful in detecting LCP in the past (Bewernitz et al., 2012). These data suggest that LCP is a common, ubiquitous phase that is expected to affect carbonate chemistry in many different CO_2_-containing aqueous systems. **(A)** Simulated Cretaceous seawater at roughly seven times atmospheric *P*_CO2_; measured DIC = 1.8 mM carbon. **(B)** Sand-filtered, modern seawater from Monterey Bay, CA; measured DIC = 1.7 mM carbon. **(C)** CarbonMix liquid, LCP solution that primarily consists of NaHCO_3_ and KCl; measured DIC = 148 mM carbon. **(D)** Fetal Bovine Serum, 1/100 diluted in deionized water and filtered through a 200 nm syringe filter; measured DIC = 0.5 mM carbon. (**Insets**) Still shots of the scattering projection used to size the LCP droplets for each respective solution.

We report here the existence of droplets of a liquid condensed phase (LCP) that are bicarbonate-rich, or *hyperalkaline*, relative to the bulk solution. The LCP droplets have been identified in ancient and modern seawater compositions (Figures 1A, B, respectively), industrial CO_2_ capture and mineralization solutions for CO_2_ sequestration (Figure 1C) and blood serum (Figure 1D). A LCP is present in simple sodium carbonate (Na_2_CO_3_) solutions over a range of pHs for HCO_3_^−^ as well as in more complex solutions from which carbonate minerals form, such as electrolyte solutions forming CaCO_3_ polymorphs similar to those seen in CaCO_3_ skeletal biomineralization or solutions containing sodium bicarbonate (NaHCO_3_) with varying ratios of Ca^2+^ and magnesium (Supplementary Figure S1). The presence of LCP requires that seawater, capture fluids in CO_2_ sequestration, blood and biomineralizing solutions be viewed as two-phase rather than one-phase systems. As a result, calculated estimates of the above-mentioned variables of these systems from two measured values do not fully constrain the system. When Ca^2+^ is present in these systems, even at near-neutral pH where HCO_3_^−^ is dominant, the HCO_3_^−^ is particularly concentrated in the hyperalkaline LCP phase, which leads to CaCO_3_ precipitation and evolution of gaseous CO_2_.

**FIGURE 2:**
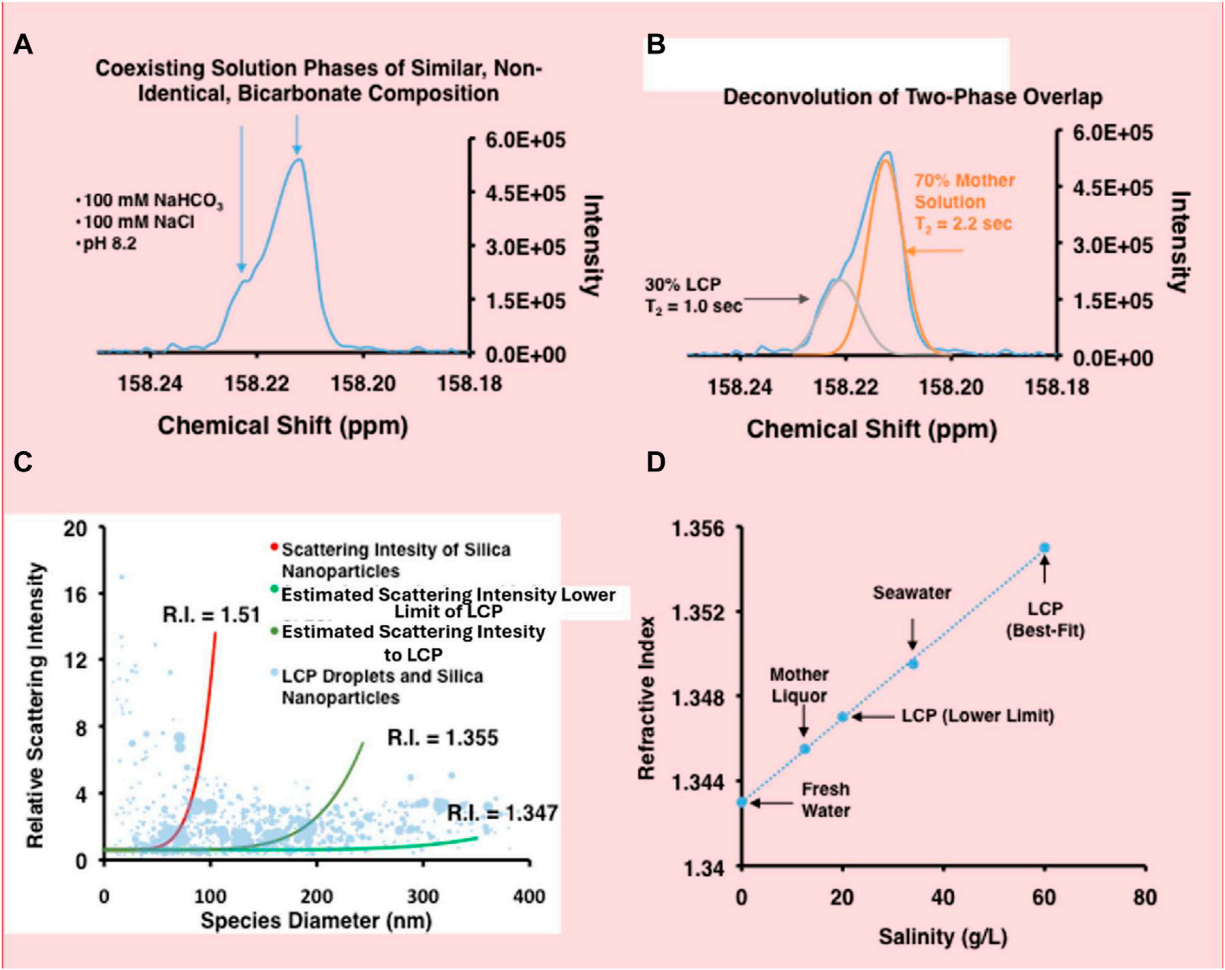
The bicarbonate-rich LCP behaves as a two-phase, solution-state system. **(A)** ^13^C NMR data of 100 mM NaH^13^CO_3_-NaCl solution. The shoulder at ∼ 158.22 ppm is attributed to the presence of LCP (Bewernitz et al., 2012), which has a composition similar to, but not identical to that of the mother solution. The small difference in chemical shift may be due to differences in pH between the LCP and the HCO_3_^−^-containing mother solution. No chemical shift standard was added to the solution to ensure that the electrolyte behavior is un-adulterated. **(B)** Deconvolution of the overlapping peaks in the ^13^C NMR spectrum in **(A)** suggests that carbon is distributed approximately 30% in LCP and 70% in the mother liquor phase. The T_2_ relaxation of the peaks was obtained through a CPMG-NMR method and determined to be 1.0 and 2.2 for the LCP and mother liquor phase, respectively, values that are consistent with solvated ions. The lower T_2_ value for the LCP is consistent with the LCP being a more concentrated, viscous solution relative to the mother solution. **(C)** The plot shows relative light scattering intensities of different sized species in solution; larger data points represent improved statistics (blue circles). SiO_2_ nanoparticles (red line, RI = 1.51) were used as a calibration standard, mid-range estimate scattering intensities of LCP droplets (dark green line, RI = 1.355) and lower-limit estimate scattering intensities of LCP droplets (green line, RI = 1.347). The RI range for the LCP droplets are consistent with saltwater solutions. **(D)** The linear relationship between saltwater salinity (g/L) and the RI is shown and is taken from literature (Quan and Fry, 1995). According to the relationship, LCP has a lower-limit salinity of approximately 20 g/L and a best-fit salinity of approximately 60 g/L.

Nuclear magnetic resonance (NMR) spectroscopy has been used in the past to characterize bicarbonate-bearing LCPs (Bewernitz et al., 2012) and early HCO_3_^−^/CO_3_^2−^ nucleation behavior (Gebauer et al., 2010) in those systems. ^13^C NMR data confirm that the LCPs are bicarbonate-rich relative to the bulk solution (Figures 2A, B). A NaHCO_3_/sodium chloride solution was characterized using one- (1D) and two-dimensional (2D) transverse relaxation measurements in order to identify and analyze the LCP, (see Figures 2A, B, respectively). The 1D relaxation measurement in Figure 2A shows a doublet in the spectrum that is consistent with the presence of two similar, yet non-identical solution states coexisting in equilibrium; LCP and mother solution. Deconvolution of the two peaks suggests that, over the course of the four-second time average used to acquire the data, approximately 30% of the inorganic carbon in solution is present as LCP. The CPMG T_2_ relaxation measurement in Figure 2B demonstrates that the two phases have different T_2_ relaxation times, re-enforcing the notion that the two peaks are due to HCO_3_^−^ residing in two distinct solution environments. Bewernitz et al. had previously used NMR in a similar fashion to support conclusions about LCP in supersaturated HCO_3_^−^ solutions containing Ca^2+^ (Bewernitz et al., 2012). The results presented in this report are significant in that they demonstrate the presence of an LCP in the absence of divalent cations and at concentrations that are undersaturated with respect to any solid phases, such as NaHCO_3_, suggesting that LCPs are not a specific step in the nucleation process, but rather an ubiquitous and fundamental electrolyte behavior occurring in solutions containing HCO_3_^−^.

Our explanation for the “shoulder” observed in the NMR spectra of HCO_3_^−^ solutions is centered on the assumption that there are two distinct chemical environments that are similar but non-identical in which HCO_3_^−^ resides. However, an alternative interpretation of the data suggests that the shoulder is due to chemical shift anisotropy (Buckingham, 1960; Carper et al., 2004). This phenomenon occurs due to the asymmetric susceptibility of the ^13^C nucleus in a static magnetic field (B_0_), and depends on the ^13^C nucleus orientation with respect to B_0_. In solution-state NMR, the differences are usually averaged out due to rapid tumbling. In solid-state NMR, the differences are strongly manifested as broad peaks that encompass all the static orientations of the NMR-sensitive nuclei. For the NaHCO_3_ solution presented in Figures 2A, B, an intermediate situation is present. According to a recent definition of the HCO_3_^−^ tensors (Stueber et al., 2002), there appears to be rapid averaging of the HCO_3_^−^ chemical shift tensors in the δ_P_ and δ_⊥_ tensors, but much less rapid averaging in the δ_a_ tensor. This situation is consistent with some ordering that occurs between HCO_3_^−^ aligning at an interface similar to amphiphilic species that make up the interface of micelles, emulsion droplets, or vesicles with the δ_P_ and δ_⊥_ tensors aligned and rotating at the interface, and the δ_a_ tensor in relative stasis (Lindstrom et al., 2006). In order to verify this possibility, however, high-resolution magic angle spinning experiments, similar to those conducted on micelles, vesicles, and liposomes, need to be carried out with the bicarbonate-rich LCP.

Light scattering measurements by nanoparticle tracking analysis (NTA) were used to calculate a first-order approximation of the refractive index (RI) of bicarbonate-rich LCP droplets (see Supplementary Figure S2). NTA has proven successful in providing estimated RIs in earlier studies (Filipe et al., 2010; Bewernitz et al., 2012). In NaHCO_3_-NaCl solution, the bicarbonate-rich LCP droplets show a low intensity, polydisperse size distribution. This suggests that the droplets have a lower RI than the mother solution and that their RI is a range rather than a single value. Figure 2C shows a best-fit to statistically relevant data points, giving a calculated RI of 1.355 for the LCP droplets. To encompass the range of the diverse RI, a lower-limit fit estimation, inclusive of all the LCP droplets, gives a RI of 1.347. Salinity of the bicarbonate-rich LCP was estimated by plotting saltwater RI vs. salinity (Quan and Fry, 1995), and is shown in Figure 2D. The values are consistent with a bicarbonate-rich LCP, having salinity between 20 and 60 g/L, that is phase-separated from a mother solution with salinity of 15 g/L. In other words, the bicarbonate-rich LCP has a salinity of four times greater (or more) compared to the mother solution. This demonstrates just how condensed the LCP can be and, to the best of our knowledge, represents the first attempt to estimate the salinity of bicarbonate-rich LCP droplets using this novel technique.

**FIGURE 3:**
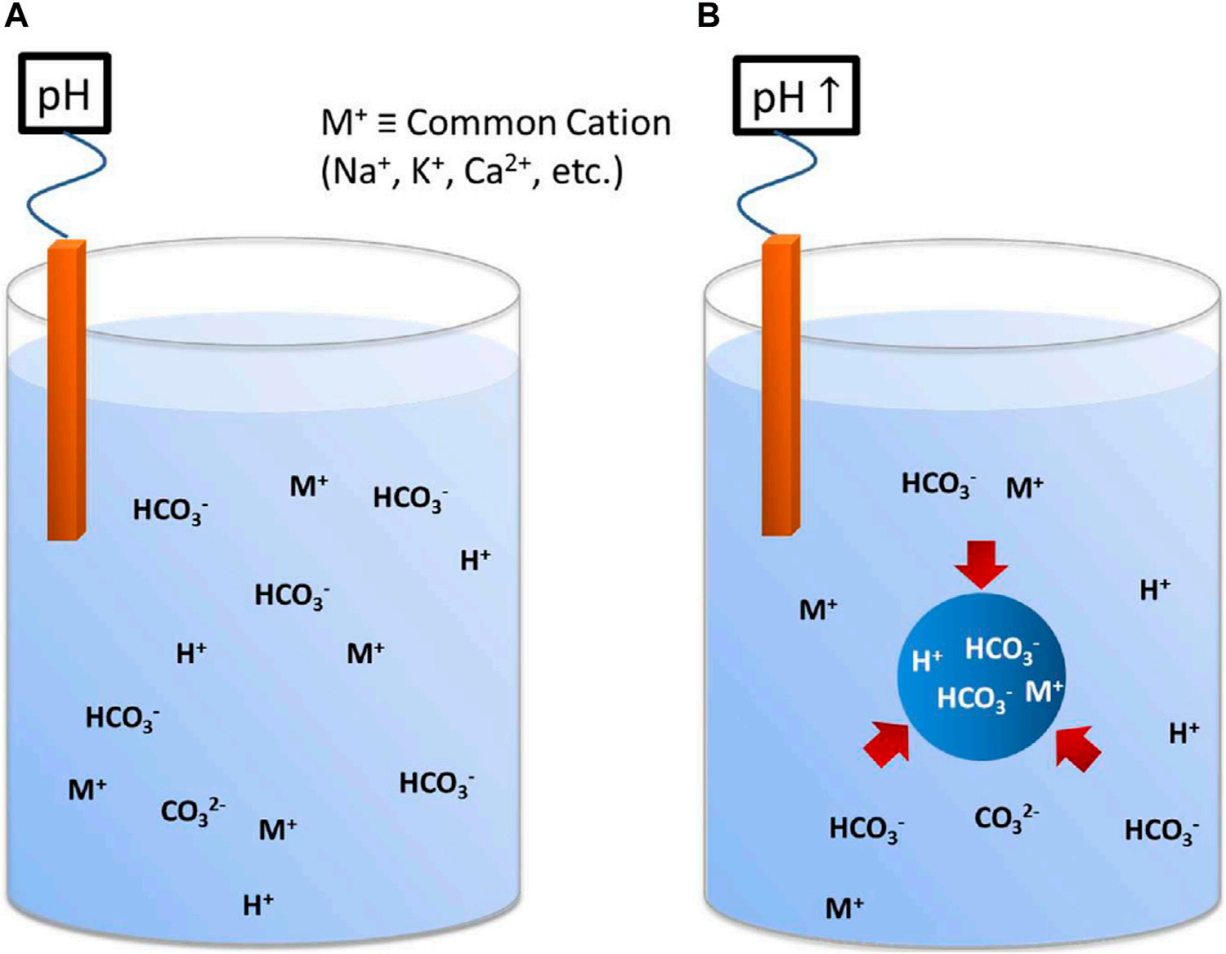
Illustration depicting how the two-phase bicarbonate-rich LCP system might alter the interpretation of system measurements such as pH. **(A)** Hypothetical one-phase system, at relatively neutral pH, that consists of HCO_3_^−^ and CO_3_^2−^ ions charge balanced with H^+^ and various M^n+^ cations, such as, Na^+^, K^+^, Ca^2+^, etc. **(B)** A system containing the same constituents as **(A)** but now they are arranged in a two-phase system that has bicarbonate-rich LCP, illustrated by the single large droplet. Even though the systems are identical in a global sense (overall DIC and alkalinity), the variables measured only in the bulk of the two-phase system, such as pH, conductivity, and selected ion concentrations, will not reflect the contents of the LCP. This can lead to misinterpretation of the system behavior unless the two-phase system is considered. For this illustration, the pH of the two-phase system would be higher due to the known sequestration of H^+^ in the LCP, leading to the false conclusion that the overall HCO_3_^−^/CO_3_^2−^ ratio has dropped when, in a global sense, it has remained constant. In a two-phase system, such as one containing bicarbonate-rich LCP, pH and alkalinity are independent due to the presence of an extra degree of freedom.

As the NMR characterization showed, the large fraction of carbon present in the LCP is consistent with the amount of “missing” HCO_3_^−^, as would be defined by the various empirically measured fitting functions and activity coefficients used to model electrolyte solutions. This suggests that the formation of LCP is part of a fundamental mechanism that has been detected in previous studies but, to this point, has not been explicitly included in electrolyte solution models. The large fraction of carbon in the two-phase LCP system (see Figure 2B) plays a significant role in the chemistry and mineral nucleation of various HCO_3_^−^-containing systems. Explicit accounting for LCP may in fact simplify our thermodynamic and kinetic models of electrolyte solutions and provide new insights into previously unexplained behaviors. Electrolyte solutions deviate from ideal behaviors by lowering the activity of individual ions. Many such mechanisms are suspected to contribute, at least in part, to the loss of activity. These include Debye-Hückel screening, ion-pairing, changes in ion hydration and solvent structure, and the recently discovered prenucleation clustering (PNC) (Gebauer et al., 2008). LCP (Bewernitz et al., 2012) is an additional phenomenon that can account for a portion, or even the majority of, the non-ideality of concentrated salt solutions, simply by lowering the ion activity due to the incorporation of ions into an LCP phase. The ions present in a separate immiscible phase do not contribute to thermodynamic measurements of the “mother solution” so would tend to lower the activities of those species (see Figure 3). We propose a new approach to modeling salt solution properties by considering the formation of a two-phase bicarbonate-rich LCP system. For additional data and experiments that correlate the LCP to non-ideal behavior, see Supplementary Figures S3, S4.

Perhaps the most significant implication of the discovery of bicarbonate-rich LCP lies in the application of carbon capture and sequestration. The ability to manipulate and isolate LCP is unlike any other approach to CO_2_ capture and utilization. Based on the mineralization process used by calcifying aquatic invertebrates that form mineralized skeletons, CO_2_ is converted to solid carbonate material via the reaction of Ca^2+^ with two equivalents of HCO_3_^−^, Eq. 1 (ΔG = −27.3 kJ mol^−1^). By carrying out the process at near-neutral pH where DIC speciation is predominantly HCO_3_^−^, relative to dissolved CO_2_ (H_2_CO_3_) and CO_3_^2−^, organisms are able to control the crystal size, shape, orientation, phase, texture and location during the deposition of CaCO_3_ biominerals.

$${\text{CaCl}}_{2}\left(\text{aq}\right)+2{\text{NaHCO}}_{3}\left(\text{aq}\right)={\text{CaCO}}_{3}\left(s\right)+{\text{CO}}_{2}\left(\text{aq}\right)+H_{2}O\left(l\right)+2\text{NaCl}\left(\text{aq}\right)$$ (1)

That the mineralization process happens at physiological pH has huge implications for large-scale CO_2_ sequestration and production of synthetic carbonate solids. In fact, we have verified, at laboratory scale, reaction conditions for Eq. 1 in the range of pH 6–8.5, producing yields of CaCO_3_ and CO_2_ of 80% and 90%, respectively (see Supplementary Figure S5). The biomimetic approach has been verified in a continuous process as well, whereby a solution of NaHCO_3_ is introduced into a solution of CaCl_2_ in a continuous flow set-up with flow-rates on the order of several liters per minute.

Typical approaches to commercial synthetic CaCO_3_ minerals involve reacting Ca^2+^ with CO_3_^2-^, Eq. 2 (ΔG = −49.87 kJ mol^−1^). This reaction occurs at roughly pH 10 due to the predominance of CO_3_^2−^, relative to H_2_CO_3_ and HCO_3_^−^. The high pH of the reaction in Eq. 2 is therefore a major limitation to prepare synthetic CaCO_3_ because of the need to increase the alkalinity in the system.

$${\text{CaCl}}_{2}\left(\text{aq}\right)+{\text{Na}}_{2}{\text{CO}}_{3}\left(\text{aq}\right)={\text{CaCO}}_{3}\left(s\right)+2\text{NaCl}\left(\text{aq}\right)$$ (2)

It is commonly assumed that CO_2_ capture and geological sequestration will provide the primary solution for managing carbon emissions on a world-wide sustainable basis. This requires that CO_2_ be in a substantially pure form as it must be compressed and liquefied for transport and injection into subsurface geological reservoirs, which will require subsequent monitoring. The most significant quantities of carbon emissions originate from Portland cement plants, coal- and natural gas-fired power plants, all of which emit dilute streams of CO_2_, but contain mainly nitrogen. Current state-of-the-art technologies to purify CO_2_ from industrial flue gas, such as amine scrubbing (Rochelle, 2009), are energy intensive primarily due to the stripping of pure CO_2_ out of the capture solution. At a coal-fired power plant, for example, purifying the CO_2_ from a flue stream can require more than 35% of the electricity generated by the plant. In the context of the reaction in Eq. 1, however, where two equivalents of CO_2_ (as HCO_3_^−^) produce one equivalent of pure CO_2_ and one equivalent of sequestered CO_2_ (as CaCO_3_ for building materials), such energy intensive loads might be circumvented.

At scale, we envision a solution to carbon capture and sequestration as a four-stage process (see process flow diagram in Supplementary Figure S6). CO_2_ capture solutions are created using an alkali recovery process driven by ion concentration gradients. Cation-selective dialysis membranes remove protons (H^+^), *i.e.*, acid, from NaCl feed solution into a draw solution, taking with it a chloride ion (Cl^−^). What is left behind is a feed solution that is higher in alkalinity. The capture solution is combined with a source of gaseous CO_2_ in a gas-liquid contactor to form HCO_3_^−^ solutions that have the propensity to separate into LCP droplets. The bicarbonate-rich LCP is separated and recovered from their bulk solution by established membrane desalination technology. Nanofiltration (NF) has proven to be especially effective. Using NF membranes we have found that the droplets do not behave like ions, but instead act like intact larger moieties that are rejected by the membrane, and therefore concentrating the LCP droplets (see Supplementary Figures S7, S8). Combination of the now concentrated bicarbonate-rich LCP with hard Ca^2+^ brine solution results in the formation of synthetic CaCO_3_ and the concomitant evolution of CO_2_, by way of the reaction in Eq. 1. If, for example, the process were fitted to a 500 MW coal-fired power plant, 9,410 tons per day (TPD) of solid CaCO_3_ mineral and 4,138 TPD pure CO_2_ would be produced, assuming 52% recovery of CO_2_ (see Supplementary Figure S9 for detailed metrics at each stage). The energy consumption lies mainly in pumping the water required for the process.

**FIGURE 4:**
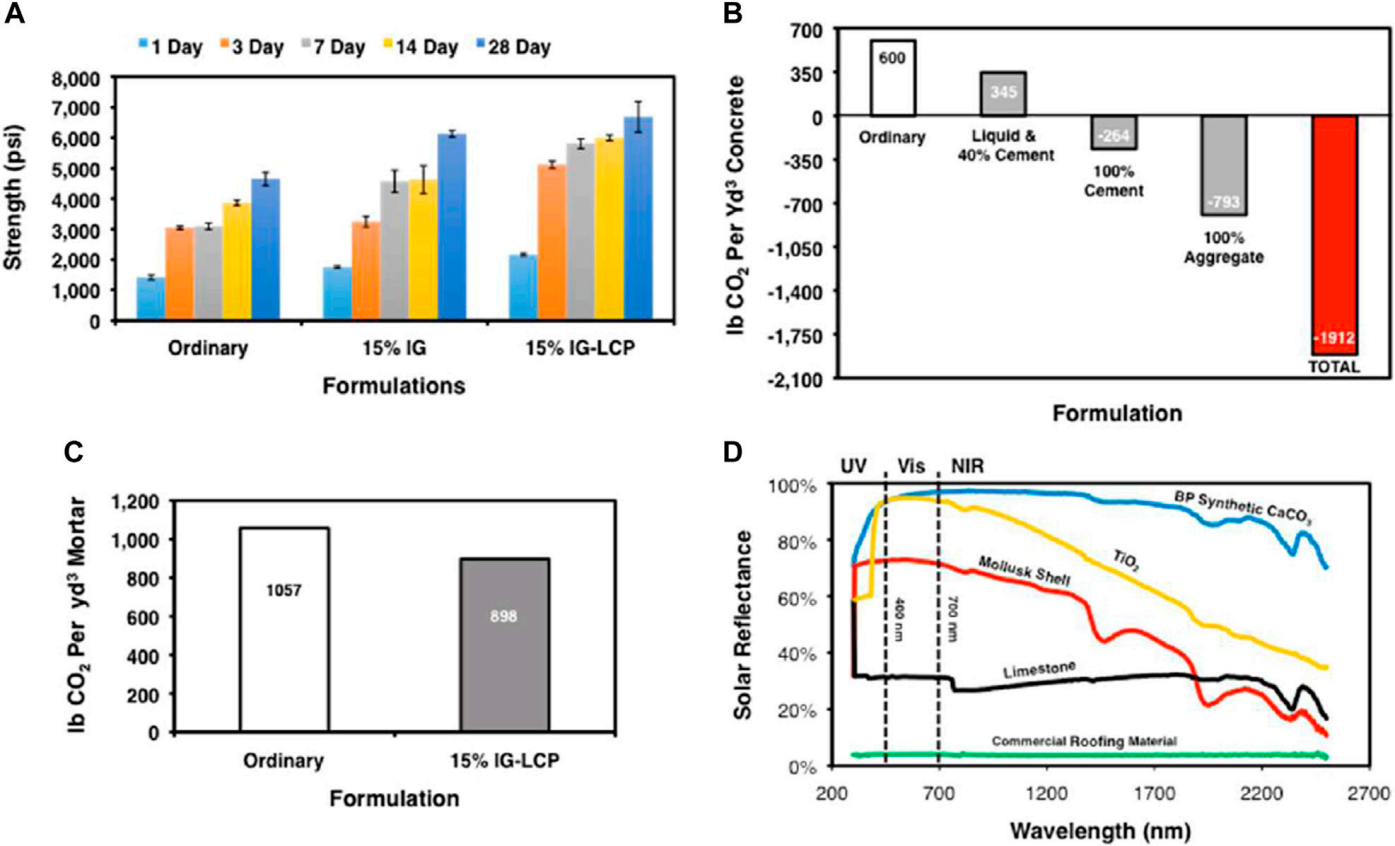
Blue Planet materials data. **(A)** Compressive strength data at 1-, 3-, 7-, 14- and 28-day for three different mortar cube formulations: Ordinary, 15% IG and 15% IG-LCP, where IG = interground limestone, LCP = CarbonMix liquid. **(B)** Life cycle analyses of different concrete formulations where traditional components are replaced by novel carbon-reducing components. The formulations are based on a generic “moderate-strength” mix design (Mehta and Monteiro, 2006). **(C)** Life cycle analyses of mortar specimens in **(A)**, where 15% OPC was replaced by quarried limestone and water was replaced by CarbonMix liquid that contained 1% CO_2_ by weight. **(D)** Solar reflectance (SR) spectra comparing Blue Planet synthetic CaCO_3_ (blue line), technical grade TiO_2_ (yellow line), *Acmaea mitra* mollusk shell (red line), Calera limestone (black line) and GAF Quickstart roofing material (green line).

Ideally, the synthetic CaCO_3_ produced in the process will be used in the built environment in the form of concrete. A formulation of water, cement and aggregate constituents, concrete is the most used building material in the world and represents the largest potential sustainable reservoir for CO_2_ sequestration. The carbon footprint of a cubic yard (yd^3^) of concrete, however, is tremendous, almost entirely due to the energy intensive process of manufacturing ordinary Portland cement (OPC) (National Ready-Mix Concrete Association, 2012). It is therefore of significant interest to replace traditional components of concrete with novel, carbon-reducing components that originate from LCP solutions. For example, Blue Planet’s CarbonMix liquid (Figure 1C) is a concentrated LCP solution that is used as a complete water replacement in concrete formulations. When used in formulations where OPC is replaced by, natural limestone, CarbonMix liquid has a significant impact on the carbon footprint per yd^3^ concrete. This includes the offset of CO_2_ that would have otherwise come from the manufacturing of OPC, as well as storage of CO_2_ sequestered in the concrete. Figure 4A shows the time-dependent compressive strength data for a series of mortar specimens that use CarbonMix liquid with a reduced OPC formulation, which has a pronounced effect on the curing properties. Other examples of carbon-reducing components for concrete include cement and aggregate replacement by synthetic CaCO_3_. These carbon-reducing components have an even bigger impact on the carbon footprint per yd^3^ concrete (Figure 4B). This is illustrated further by the abovementioned mortar specimens, which have a lower carbon footprint the ordinary mortar specimen formulation (Figure 4C).

The carbonate minerals produced from LCP solutions are not only valuable to concrete, but we’ve also found that minerals precipitated via the reaction in Eq. 1 exhibit unusually high solar reflectance (SR) relative to known commercial and natural materials (Figure 4D). High SR is desirable for commercial cool roofing technologies as a means to mitigate heat island effects in urban areas (Akbari et al., 2009; Santamouris et al., 2011), reducing the convective and radiative thermal gain of a surface.

In the field of biomineralization, it has long been proposed that inorganic carbonates can be induced, through biological additives, to condense into a concentrated liquid phase just prior to mineral formation, specifically CaCO_3_ (Gower and Odom, 2000; Faatz et al., 2004; Gower, 2008). The presence of charged organic moieties, such as those in seawater and in serum, appear to have a profound affect on the coalescence and abundance of the LCP droplets, and are known to be associated with biomineralization processes. For example, the putative role of the acidic glycoproteins that are ubiquitous in all carbonate and phosphate mineralized tissues has been proposed to be in mineral nucleation, crystal growth modulation and calcium transport. Given the strong influence that organic polymers in seawater and serum appear to have on the size and abundance of LCP, LCP-modulation may represent a functional role for these organic moieties, and explain their intracrystalline presence in these skeletal materials. Many CaCO_3_ biomineralization processes have been difficult to explain with single-phase models. It has been assumed that pre-nucleation phases possess “CaCO_3_” stoichiometry and not HCO_3_^−^ stoichiometry. Here, we find that the initial stoichiometry may exist as a calcium bicarbonate association with LCP, especially in the presence of biological agents. We provide evidence that the bicarbonate-rich LCP does not require supersaturated conditions to form and is neither mineral specific nor transient, but rather, is a fundamental electrolyte behavior exhibited by HCO_3_^−^, which exists at undersaturated and apparent equilibrium conditions. The resulting potential for additional control over the bicarbonate-rich LCP droplets has far-reaching ramifications in such fields as oceanography, environmental sciences (Lee et al., 2010), material sciences and carbon sequestration. Long-lived, stable LCP droplets at undersaturated conditions allow for a previously unknown means to manipulate solvated inorganic carbon chemistry, such as mechanical separation, concentration and storage of inorganic carbon in the solution state, and the engineering of synthetic minerals with superior properties.

## Data Availability

The original contributions presented in the study are included in the article/Supplementary Material, further inquiries can be directed to the corresponding authors.
