# Supplementary material for: Discovery of a hyperalkaline liquid condensed phase: significance toward applications in carbon dioxide sequestration
Source: Front Bioeng Biotechnol. 2024 Apr 30;12:1382071. doi: 10.3389/fbioe.2024.1382071 (PMC11091406; doi:10.3389/fbioe.2024.1382071)
Supplement: Supplementary file 1 [file DataSheet1.pdf]

Supplementary Materials for

Discovery of a Hyperalkaline Liquid Condensed Phase: Significance Toward Applications in Carbon Dioxide Sequestration

Mark A. Bewernitz*, Jacob Schneider, Christopher L. Camiré, Seung-Hee Kang, William L. Bourcier, Richard Wade, and Brent R. Constantz*

*Correspondence to: bewernitzmark@gmail.com, brent@blueplanetsystems.com; www.blueplanetsystems.com

Materials and Methods

NTA image collection

Data was collected on a NS-500 nanoparticle tracking analyzer (Malvern) due to its capabilities to analyze dilute species in solution in a way that dynamic light scattering cannot (*1*). Raw data consisted of 60 second recordings of the scattering projections of various solution sat a camera length setting of 16. Still shots used in figures are representative still shots of this recording. The recording was processed to generate the histogram of particle size vs. particle count with a bin size of 20 nm. The settings used to process the raw data were a detection threshold of 6, auto-blur, auto-minimum expected particle size, and a solution viscosity equal to that of water at 25 °C (0.89cP).

Sample Preparations for NTA image collection

The simulated Cretaceous seawater at a pCO_2 simulating the cretaceous era seen in Figure 1A was created by mixing 23.9 g NaCl (Sigma-Aldrich, S9888), 4.01 g Na_2SO_4, anhydrous (Acros Organics, S421), 0.68 g KCl (Sigma Aldrich, P9541), 0.2 g $NaHCO_3$,(Aqua Solutions, 144-55-8), 3 g $MgCl_2$ hexahydrate (Research Products International Corp., M24000), and 3.5 g $CaCl_2$ dihydrate (Aqua Solutions, C0630) into 1 liter of nanopure water to create a simulated Cretaceous era seawater (1:1 $[Mg^{2+}]/[Ca^{2+}]$ ratio). The solution was titrated to pH 8.1 with NaOH (aq) (Alfa Aesar, A 18395) and then gassed with CO_2 to pH 7.7 to simulate the higher P_{CO2} of the Cretaceous era. The value, pH 7.7 was chosen by using Geochemist's Workbench™ to estimate the pH of the saltwater solution in equilibrium with a P_{CO2} of 7 times that of the modern era prior to the industrial revolution (210 ppm). Modern seawater (seen in Figure 1B) was obtained from Monterey Bay, California and was sand-filtered at the Monterey Bay Aquarium Institute at Moss Landing, California. The simple bicarbonate solution seen in Figure 1C consisted of 100 mM $NaHCO_3$ and 100 mM NaCl. The control with which it was compared in Figure S2 consisted of a 200 mM NaCl solution. Adult Fetal Bovine serum was analyzed by using Adult Bovine Serum (Sigma B9433, batch 12B519) and diluting 1:100 in nanopure water prior to filtering with a 200 nm diameter pore size syringe filter (Whatman Cat. No. 6809-1122).

Sample Preparation NMR Measurements

A solution containing 100 mM $NaHCO_3$ and 100 mM NaCl was created using 100% ^{13}C substituted $NaHCO_3$ (Cambridge Isotopes, 372382). No chemical shift standard was added to the solution to ensure electrolyte behavior is naturally occurring. All NMR data was obtained using a

Varian Inova 500 magnet operating at 126 MHz using a 5 mm broadband probe. All experiments were conducted at 298 Kelvin. Deuterium oxide (Aldrich, 151882) was used to obtain a lock at a volume fraction of 2.5% of the total sample. Data was processed using NUTS™ and Microsoft Excel software when deconvolution of overlapping spectral peaks was required. 90° pulses were used with acquisition times of 6.35 seconds. The T_2 relaxation measurements were conducted using a Carr-Purcell-Meibloom-Gill (CPMG) sequence with increasing tau (τ) times of 0.025, 0.05, 0.1, 0.2, 0.4, 0.8, 1.6, 3.2, 6.4 seconds.

Refractive Index measurement

Species in the size range of the bicarbonate-rich LCP are generally in the Rayleigh scattering regime, where a close approximation of the efficiency of light scattering is given by equation (S1) below:

$$I \propto \sigma_s = \frac{2\pi^5}{3}\frac{d^6}{\lambda^4}\left(\frac{n^2-1}{n^2+2}\right)^2 \qquad (1)$$

Where:

I is the measured intensity of the scattering event

σ_s is the scattering cross section

d is the species diameter

λ is the wavelength of incident light, here λ = 402 nm

n is the ratio of the scattering species refractive index to the solvent refractive index

Equation (S1) relates the measured intensity of a scattering event (I) to the diameter of the scattering center species (d), the wavelength of incident light (λ) and the refractive indices (RI) of the scattering species and solvent. The NTA technique measures I directly and d by means of Brownian motion. If the RI of the solvent is known, a standard with a known RI can be used to calibrate the technique to account for software and equipment measurements of the relative intensity. Equation (S1) can then be used to calculate the RI for the scattering species (*2, 3*).

To determine the RI of bicarbonate-rich LCP, silica (SiO_2) nanoparticles of 50, 80, 100 nm (hydrated diameters of 62, 92, 116 nm, respectively) were used as the standard reference material (RI = 1.51). Equal masses of the different sized SiO_2 nanoparticles were placed into a solution of 100 mM Na_2CO_3 to the concentration of approximately 10^8 particles/ml. The standard solution was then titrated with 1 N HCl, to pH 9.0, and analyzed for light scattering events using NTA. Figure S2 displays the standard curve. This standard curve was applied to the data shown in Figure 2C to demonstrate the reliability of the technique and to verify and validate the technique.

Pitzer Modeling

We carried out thermodynamic calculations of ion activities in carbonate solutions using Geochemist's Workbench software (GWB). The plot in Figure S4-A was obtained by subtracting

the ion activity predicted by Pizer's Equations from the ion activity predicted by Debye-Hückel (D-H) theory. D-H theory accurately predicts ion activities up to about 10 mMolal concentration. Our assumption is that non-ideality at higher concentrations may be in part due to formation of LCP which removes some ions from bulk solution and thus lowers their activities. The sum of the deviations from D-H activities shown in S4-B are simply the sum of the deviations for all ions and approximate the percent of ions present as LCP (assuming it is responsible for the lowered ion activities). We then compare trends in ion activities vs. pH for our calculated percent of ions present in LCP with observations of the volume of LCP based on light scattering measurements of real carbonate solutions (Figure S4-C).

Concentration of bicarbonate-rich LCP by Membrane Processes

Twenty gallons of solution of LCP droplets was concentrated with a DK (GE Osmonics) NF membrane and contained dissolved ions of sodium (Na^+), potassium (K^+), chloride (Cl^-) and bicarbonate (HCO_3^-); conductivity = 10.8 mS. The NF system houses two custom made 1.8 × 12 inch elements, purchased from Membrane Development Specialists (Solana Beach, CA), and is operated by a positive displacement pump capable of moving roughly 2 gal water per minute. The NF system was operated at the flow rate specified by the membrane manufacturer. The feed solution was concentrated by continuously recirculating the concentrate back into the feed and removing permeate in incremental volumes. Potassium chloride (Sigma Aldrich) and sodium bicarbonate (Aqua Solutions, Sigma Aldrich, Church & Dwight Co. Inc.) were purchased commercially and were used without further purification.

Rejection of $NaHCO_3$ by various membrane types - reverse osmosis (RO), X20 (TriSep); nanofiltration (NF), DK (GE Osmonics); ultrafiltration (UF), MPF-36 (Koch Membrane Systems) - were investigated with a custom made flat-plate casing that houses 4-inch diameter sample elements, purchased from Membrane Development Specialists (Solana Beach, CA). The flat-plate system uses a positive displacement pump to move roughly 2 gal water per minute. Here, back-pressure on the system is adjusted so that permeate flow rate - in gallons of permeate per square foot of membrane per day (GFD) - meet the manufacturers specification. The Ion Rejection was calculated by dividing the conductivity of the permeate by the conductivity of the feed solution. Sodium bicarbonate (Aqua Solutions, Sigma Aldrich, Church & Dwight Co. Inc.) was purchased commercially and was used without further purification.

Preparation of Mortar Specimens

The mortar cube specimens were mixed according to ASTM C109/C109M (*4*) and used variations of the following components: Ottowa silica sand (ELE International), 16-200 mesh glass grade limestone sand (Blue Mountain Mineral, Colombia, CA), Basalite Type II-V cement, 15% interground (IG) cement, water, and liquid condensed phase (LCP) liquid. The 15% interground cement was prepared by blending 325 µm-sized natural limestone (Blue Mountain Minerals, Columbia, CA) with the Basalite cement and was then milled for 16 hours (PTA-02 Ball Mill with 10 L Jar). Mortar components were blended in a Hobart Mixer (Hobart Inc, Troy OH) according to ASTM C305 (*5*). Mortar was transferred to 50 mm × 50 mm cubic brass molds, specified by ASTM C109/C109M. Samples set in 100% relative humidity (RH), 73°F, were demolded at 1 day and further stored at 100% RH, 73°F, until mechanically loaded. Samples were mechanically

loaded at 200 lb/s on an ELE load frame (Accu-Tek Touch 250 Series) in accordance with ASTM C109/C109M.

FTIR analyses and sample preparation

FTIR spectra were recorded using a Nicolet IS-10 by Thermo-Fisher with a HeNe laser and a fast recovery deuterated triglycerine sulfate (DTGS) detector. Scans were collected on a Germanium ATR crystal at resolution of 16 and at optical velocity of 0.4747. FTIR samples were prepared by adding 0.25 M $CaCl_2$ (Sigma, Lot#BCBL2738 & Deionized Water) to 0.5M $NaHCO_3$ (Aqua Solutions, Lot #319302 & Deionized Water) (Figure S7). 20 µl was pipetted onto the ATR crystal and the reaction was recorded in a time resolved fashion using a Macro applied to Omnic 9.2 software. The spectra were recorded at 0, 10, 20, and 1800 seconds.

Dissolved inorganic carbon (DIC) analysis

The dissolved inorganic carbon (DIC) content of solution and solid carbonate samples were determined by acidometric titration and coulometric detection using a CM150 carbon analysis system (UIC, Inc.). The samples were typically titrated with 2N H_2PO_4 (Sigma Aldrich). To detect CO_2 evolved in reactions of $CaCl_2$ (Sigma Aldrich) with $NaHCO_3$ (Aqua Solutions), however, the samples were not titrated with H_2PO_4, but rather, a solution of $CaCl_2$ was titrated with a solution of $NaHCO_3$ because titration with H_2PO_4 would result in liberation of CO_2 from $CaCO_3$. This allowed CO_2 to be quantified by coulometric detection; any solid formed in the reaction was then isolated, dried and analyzed by FTIR to confirm its composition as $CaCO_3$. All analyses using the CM150 system were completed at 40 °C.

Time-resolved pH measurement

The pH was recorded in a time resolved manner using an OrionStar A215 pH meter with an Orion 8157BNUMD Ross Ultra pH/ATC Probe. Data was logged using StarCom 1.0 sampling every 3 seconds while dosing 0.25 M $CaCl_2$ solution (Sigma, Lot#BCBL2738 & Deionized Water) into 0.5 M $NaHCO_3$ solution (Aqua Solutions, Lot#319302 & Deionized Water) and 0.5 M Na_2CO_3 (Sigma Lot#SLBD98664)(Figures S7-G and S7-H)

Synthesis of Calcium Carbonates Materials with Desirable Properties

Carbonate was produced by mixing CaCl2 (aq) and NaHCO3 (aq) at a molar ratio of 1:2, similar to what is described in the Timer-resolved pH measurement section of the Material and Methods. The precipitate was then pressurized using a carver press to 20,000 lb_f and allowed it to dwell for 30 minutes. The compact calcium carbonate was then placed in a humidity chamber (Fisher Scientific Isotemp Oven Model 615F, made 100% humid with water) for 7 days at 40 °C. Finally, the sample was cured for 12 days in 1M Na_2CO_3 solution, which was kept in a water bath to maintain the temperature at 40°C.

Solar reflectance measurements and calculations

Solar reflectance spectra were collected using Perkin-Elmer Lambda 950 UV-Vis-NIR spectrometer loaded with 150 mm Integrating Sphere. The data was recorded with 5 nm interval using UV Winlab 6.0.2 software. The solar reflectance was calculated based on clear sky Air Mass 1 Global Horizontal (AM1GH) (*6, 7*) and ASTM Standard E892-87 terrestrial solar irradiance (*8*) to compute solar (averaged over range 300-2500 nm), UV (averaged over range 300-400 nm), visible (averaged over range 400-700 nm), and near-infrared (averaged over range 700-2500 nm) reflectance.

Life Cycle analysis

Calculations of lb CO_2/yd^3 mortar were based on the assumption that an average of 2,044 lb of CO_2 is emitted for every 2,205 lb of Ordinary Portland Cement (OPC) produced in the U.S., depending on fuel type, raw ingredients, and the energy efficiency of the cement plant (*9*) (essentially 1:1 CO_2:OPC produced). Therefore, ASTM C109/C109M mortar cube mix design has 1,057 lb CO_2/yd^3 mortar (extrapolated from mix design detailed in Figure 4B); this also assumes no CO_2 contributions from the water and aggregate components of the mix design. When a carbon-reducing LCP liquid that contains 1 wt% CO_2 is used as a complete water replacement in the Ordinary mix design, the lb CO_2/yd^3 mortar is reduced by 5 lb CO_2. When the carbon-reducing liquid is used in combination with a 15% replacement of OPC by interground (IG) limestone, the lb CO_2/yd^3 mortar is reduced to 898 lb CO_2/yd^3 (from 1057 lb CO_2/yd^3). This is due to the 1 wt% CO_2 in the liquid and the 15% offset of CO_2 that would have otherwise come from the manufacturing of OPC.

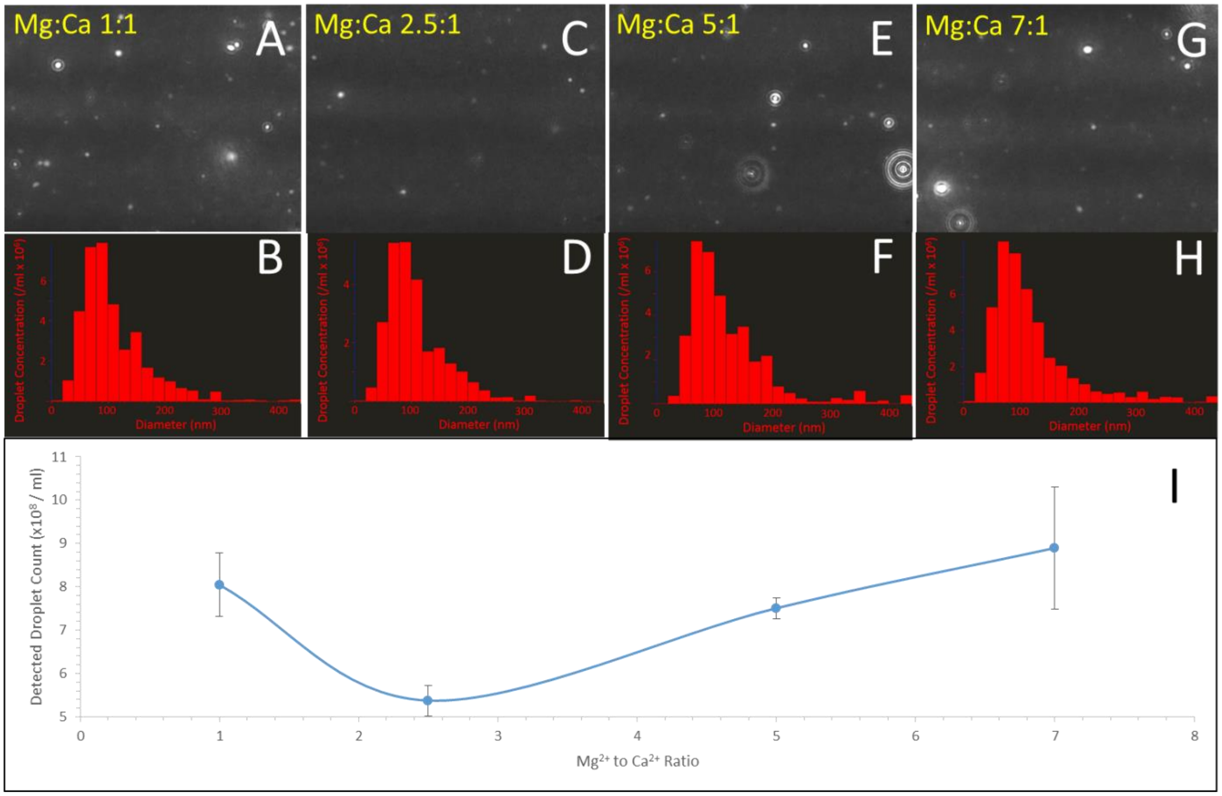

Fig. S1. The Mg/Ca ratio affects bicarbonate-rich liquid condensed phase droplets. The effect of changing [Mg^{2+}]:[Ca^{2+}] ratio to the formation of bicarbonate-rich liquid condensed phase (LCP) droplets was measured by means of nanoparticle tracking analysis (NTA) in solutions containing 0.2 mM divalent ions and 50 mM sodium bicarbonate. (**A, C, E, G**) A still-shot of the scattering projection of solutions containing [Mg^{2+}]:[Ca^{2+}] ratios of 1:1, 2.5:1, 5:1, and 7:1, respectively. (**B, D, F, and H**) The size histograms of the LCP droplets obtained by NTA for A, C, E, and G, respectively. (**I**) The LCP droplet count as detected by the nanoparticle tracking analyzer vs. the [Mg^{2+}]:[Ca^{2+}] ratio in solution. The data was collected in triplicate and averaged with a standard deviation of error in either direction. We see that the presence of divalent ions seems to promote the formation of large, robust and detectable LCP as compared to solutions containing only monovalent ions (see Fig. 1C). The size distribution of the droplets is very similar for all cases; however, the amount of LCP droplets seems to change depending on the [Mg^{2+}]:[Ca^{2+}] ratio as shown in I. At solutions near 1:1 ratio (similar to Cretaceous seawater), there are many droplets, but as the solution increases in [Mg^{2+}]:[Ca^{2+}] ratio, the number of droplets drops suddenly and then increases until, at a [Mg^{2+}]:[Ca^{2+}] ratio of 7:1 (similar to modern seawater). This effect on the LCP by [Mg^{2+}]:[Ca^{2+}] may have a role in describing ocean acidification, explaining the extreme calcium carbonate deposition during the Cretaceous era, and even explaining why calcite forms near 1:1 ratios, but aragonite forms at higher ratios (5:1 and 7:1, shown on the plot in Fig. S1I).

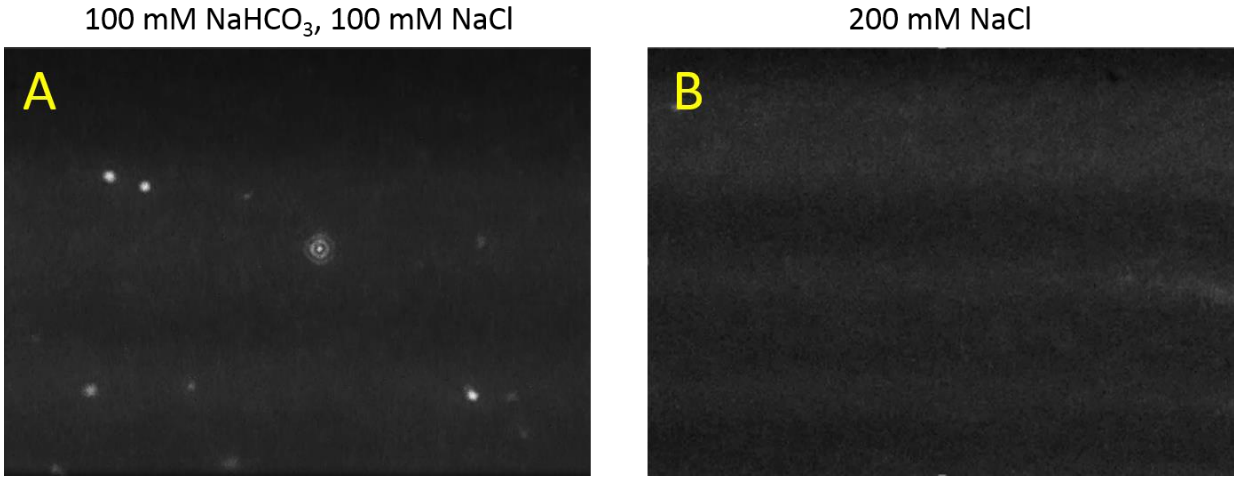

Figure S2. Bicarbonate ions form bicarbonate-rich liquid condensed phase. Still-shots of the scattering projections obtained by nanoparticle tracking analysis (NTA) strongly suggest that the bicarbonate ion participates in a condensation, as reported for bicarbonate-rich LCP. A) A solution containing 100 mM NaHCO3 and 100 mM NaCl contain many scattering events presumably due to the formation of bicarbonate-rich LCP. B) A solution containing 200 mM NaCl does not display scattering events at the same conditions. This is evidence that the bicarbonate ion participates in a condensation to form bicarbonate-rich LCP even in relatively simple, undersaturated solutions. The species seen in A are a distribution of sizes centered around 50-60 nm in diameter as shown in Figure 1C of the main report.

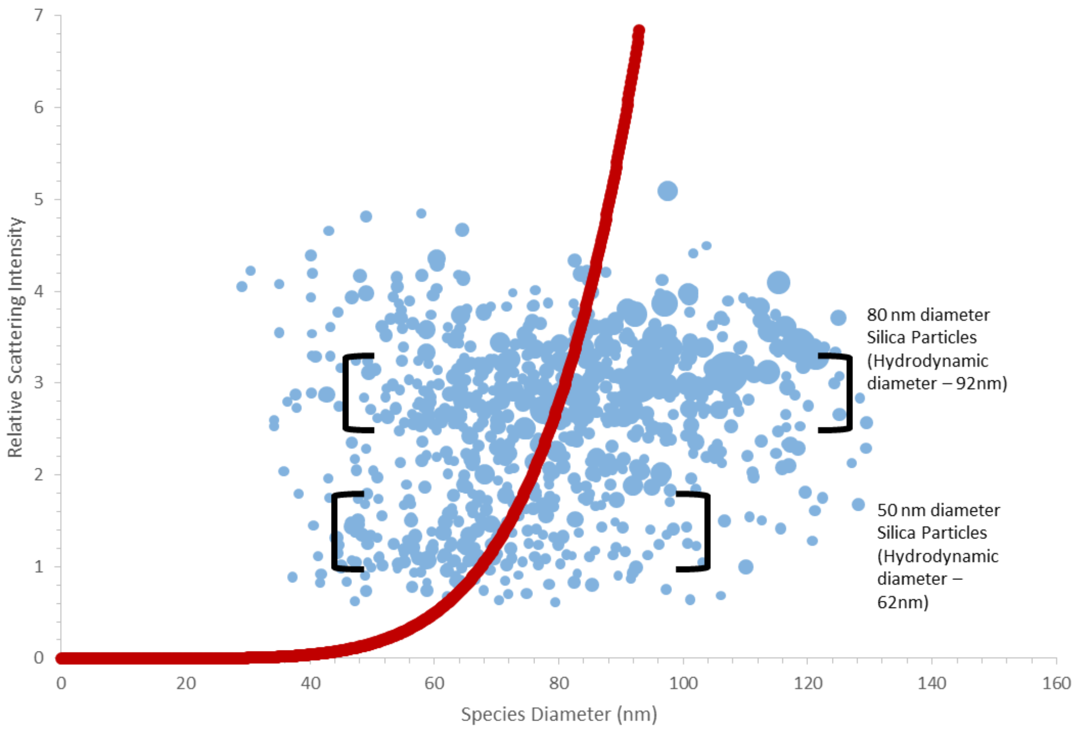

Fig. S3. The standardization of nanoparticle tracking analysis with silica particles for refractive index measurements. Scattering intensity vs. diameter of silica nanoparticles in water obtained from the NS500 nanoparticle tracking analyzer. To establish a standard curve and calibrate the NS500 NTA for refractive indices measurements, silica (SiO_2) nanoparticles (nanoComposix) of 50, and 80 nm (hydrated diameters of 62, and 92 nm, respectively) were used as the standard reference material (RI = 1.51). A curve was estimated using the Raleigh approximation (equation S1) to fit the intensity of the scattering events with the measured size of the particles. The area of the data points (blue circles) represents the relative statistical certainty of the measurement.

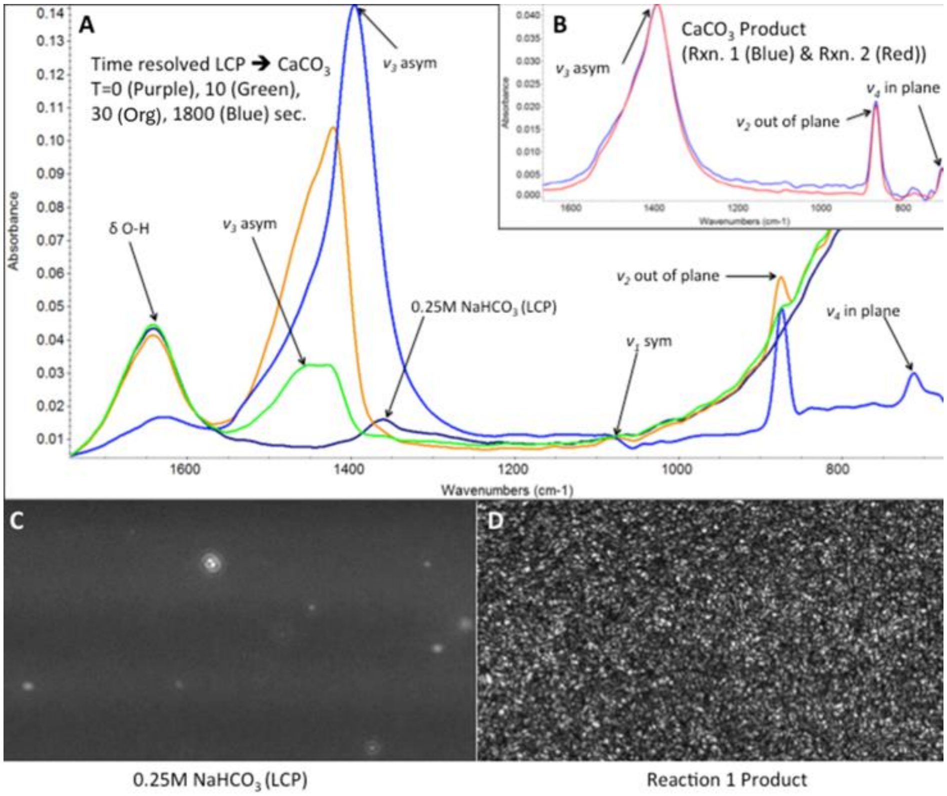

E Reaction 1: $CaCl_2\,(aq)+2NaHCO_3\,(aq) \leftrightarrow CaCO_3\,(s)+CO_2\,(g)+H_2O+2NaCl(aq)$

Reaction 2: $CaCl_2(aq)+Na_2CO_3(aq) \leftrightarrow CaCO_3+2NaCl(aq)$

F

	Rxn. Yield CO_2	Rxn. Yield $CaCO_3$
Reaction 1	90%	80%
Reaction 2	Not Expected	91%

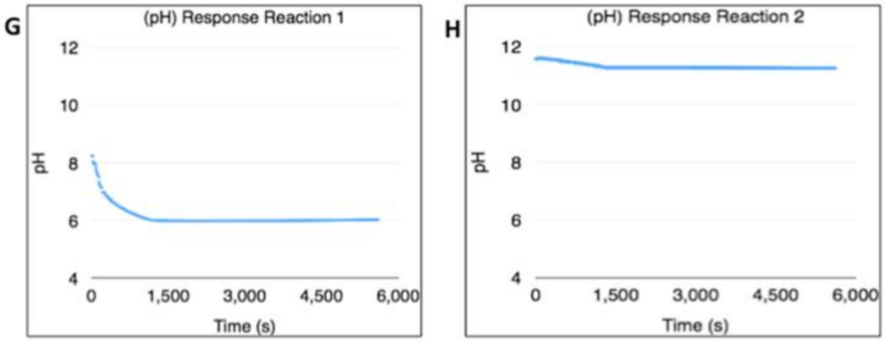

Fig. S4. Two pathways to calcium carbonate formation; a high pH pathway and a low pH pathway. 0.25 M $CaCl_2$ was added to equal volumes of either 0.5 M $NaHCO_3$ or 0.5 M Na_2CO_3 in a dump reaction manner and were analyzed immediately post mixing. The results suggest that there are two distinct pathways toward calcium carbonate formation; a familiar one designated in the main text as reaction 2 ($CaCl_2$ (aq) and Na_2CO_3 (aq) at high pH, carbonate pathway) and another pathway designated in the main text as reaction 1 ($CaCl_2$ (aq) into $NaHCO_3$ (aq) at neutral pH, bicarbonate pathway). (**A**) A Time Resolved

Fourier Transform Infrared Spectra (FTIR) of a reaction 1 dump reaction at times of 0 seconds (purple), 10 seconds (green), 30 seconds (red), 30 minutes (blue) post mixing. Calcite infrared active bond vibrational modes of, v3 (1400 cm-1), v1 (1087 cm-1), v2 (877 cm-1), and v4 (714 cm-1) are seen. The asymmetrical C-O stretching of the carbonate bond, v3, is seen shifting through a bidentate, resulting in a characteristic calcite peak suggesting that calcium carbonate formation may be forming through a bicarbonate pathway similar to one proposed in nature (*10*). The symmetric carbonate vibrational mode, v1, relates to free carbonate available in the structure. Out of plane bending, v2, and in plane bending, v4, are identified by (877 cm-1) and (714 cm-1) respectively. (**B**) A FTIR spectra identifying $CaCO_3$ (calcite) formed by LCP Reaction 1, and Reaction 2. The end product of both pathways appears to be identical. (**C**) A nanoparticle tracking analysis (NTA) still-shot image of 0.25M $NaHCO_3$. Bicarbonate-rich liquid condensed phase droplets can be seen. (**D**) A NTA still-shot image of a reaction 1immediately post mixing provides a visualization of what is measured in time-resolve fashion in part A. (**E**) The chemical pathway of LCP-driven low pH reaction (Reaction 1) vs. conventional high pH reaction (Reaction 2)., (**F**) The measured yields of reaction 1 vs. reaction 2, with respect to $CaCO_3$ and CO_2, as determined by DIC analysis. The results reinforce the difference between reaction 1 and reaction 2 pathways due to differences in evolved CO2 (expected for reaction 1). (**G**) The time-resolved pH response of reaction 1 dump reaction shows an initial drop in pH, presumably due to removal of bicarbonate. (**H**) The time-resolved pH response of reaction 2 dump reaction shows little pH drop suggesting that carbonates are being consumed during mineral formation and are buffered by bicarbonates. During the reaction of carbonate formation, liquid condensed phases (LCP) evolve in the presence of calcium ion and nucleating to form $CaCO_3$. As $CaCO_3$ precipitation proceeds, dehydration of the reaction product occurs as seen by the drop of δ O-H vibrational peak. According to FTIR spectra in Figure S5A, the structures were initially hydrated and amorphous as reported previously, showing broad peaks in the observed range (*11, 12*). As the reaction progresses, however, gradual appearance of sharp peaks are related to the development of crystalline structure of the carbonate polymorphs as seen with the increase of 1400 cm^{-1} (ν_3 asymmetrical CO_3), 1087 cm^{-1} (ν_1 symmetrical CO_3), 877 cm^{-1} (ν_2 out-of-plane band of CO_3), and 714 cm^{-1} (ν_4 in-plane-band of CO_3) (*13*), indicating the formation of calcite phase (*14*). This particular reaction was denoted as Reaction 1 in the main report and was compared to conventional $CaCO_3$ precipitation pathway, Reaction 2.

Reaction 1: $CaCl_2$ (aq) + $2NaHCO_3$ (aq) ↔ $CaCO_3$(s) + 2NaCl (aq) + H_2O (l) + CO_2 (g)

Reaction 2: $CaCl_2$ (aq) + Na_2CO_3 (aq) ↔ $CaCO_3$(s) + 2NaCl (aq)

The products as the result of Reaction 1 and 2 are identical as shown in Figure S5B. The yield of CO_2 and $CaCO_3$ were 90% and 80%, respectively, confirming the stoichiometry and chemical pathway of Reaction 1. pH was also measured in a time-resolved fashion and suggests that reaction 1 occurs at a lower pH compared to the conventional Reaction 2. This is directly related to LCP-formation mechanism as Ca^{2+} has the propensity to interact with HCO_3^-, enabling precipitation reaction to take place at neutral pH. In both cases, pHs in the initial stages decrease slightly due to onset of $CaCO_3$ precipitation.

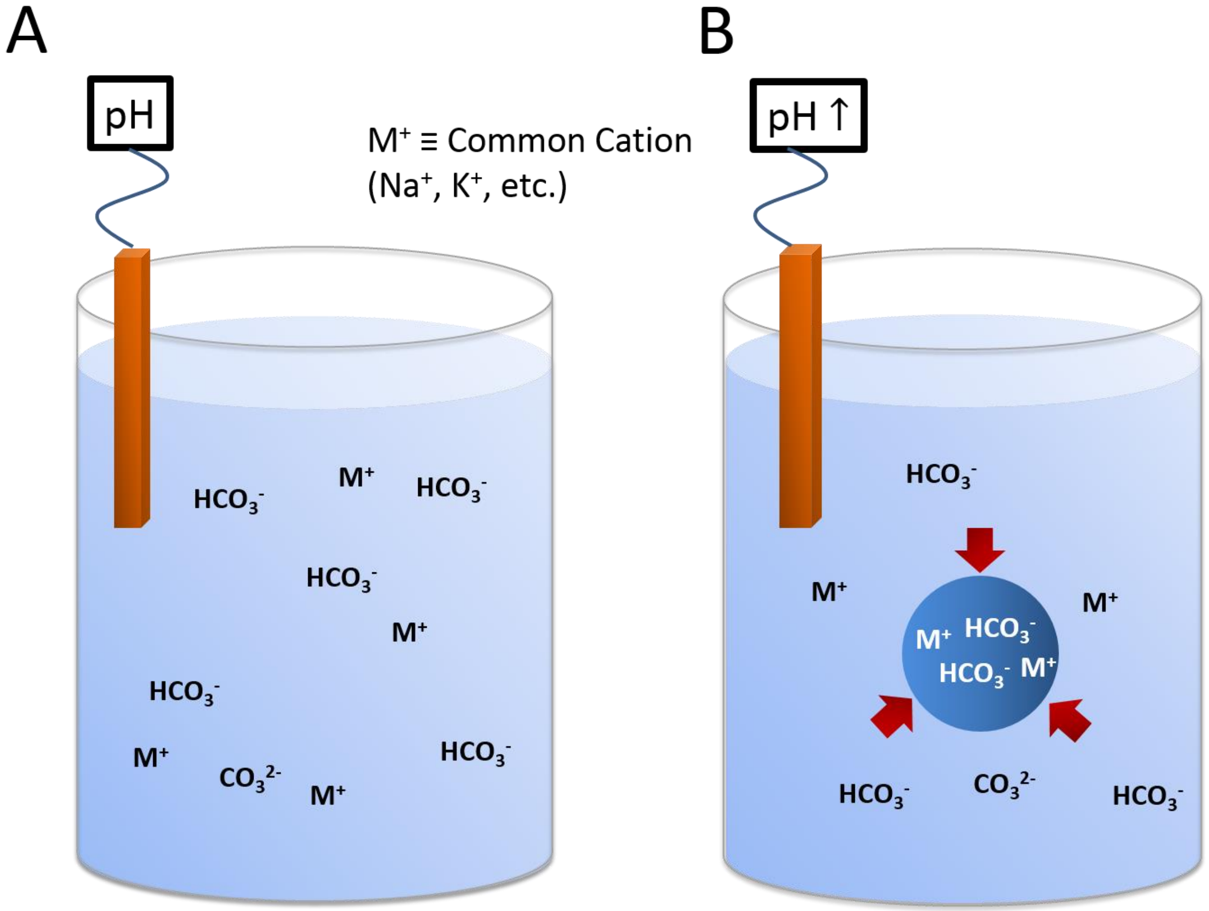

Fig. S5. Illustration depicting how the two-phase bicarbonate-rich LCP system might alter the interpretation of system measurements such as pH. (A) Hypothetical one-phase system, at relatively neutral pH, that consists of HCO_3^- and CO_3^{2-} ions charge balanced with H^+ and various M^{n+} cations, e.g., Na^+, K^+, Ca^{2+}, etc. (**B**) A system containing the same constituents as (A) but now they are arranged in a two-phase system that has bicarbonate-rich LCP, illustrated by the single large droplet. Even though the systems are identical in a global sense (overall DIC and alkalinity), the variables measured only in the bulk of the two-phase system, such as pH, conductivity, and selected ion concentrations, will not reflect the contents of the LCP. This can lead to misinterpretation of the system behavior unless the two-phase system is considered. For this illustration, the pH of the two-phase system would be higher due to the known sequestration of H^+ in the LCP, leading to the false conclusion that the overall HCO_3^-/CO_3^{2-} ratio has dropped when, in a global sense, it has remained constant. In a two-phase system, such as one containing bicarbonate-rich LCP, pH and alkalinity are independent due to the presence of an extra degree of freedom.

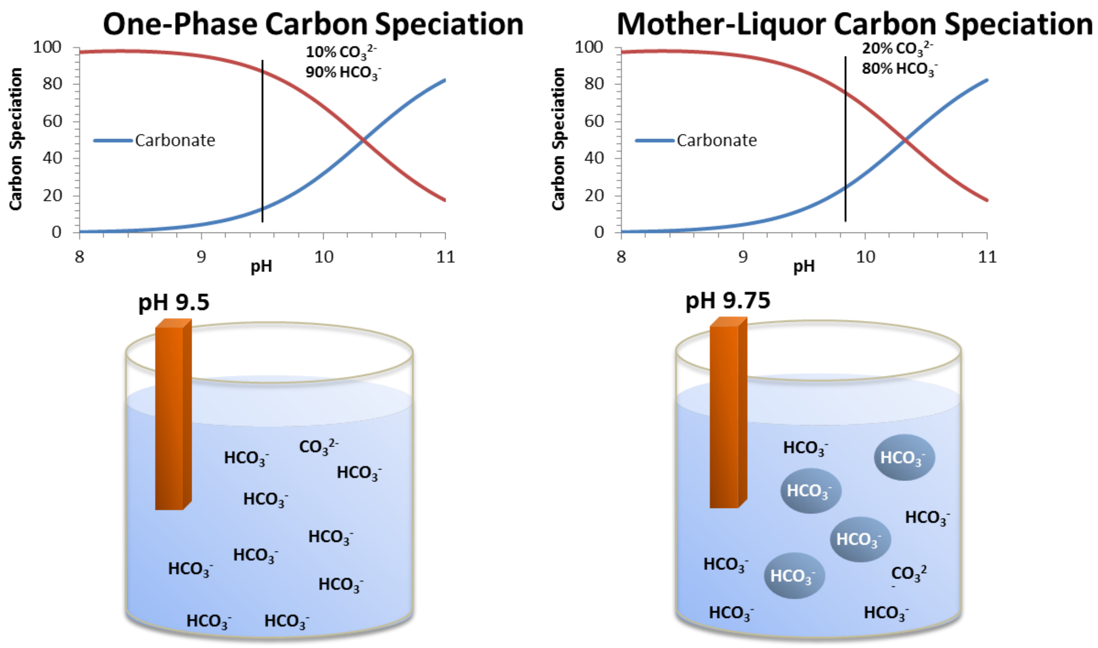

Fig. S6. An illustration of a two-phase system that can alter the interpretation of system measurements such as pH. On the left is a hypothetical one phase system which shows speciation ideally to yield a 1:10 carbonate to bicarbonate ratio. The system on the right contains the same constituents as on the left but it is arranged in a two-phase system containing bicarbonate-rich liquid condensed phase droplets within a mother liquor which is at a carbonate-to-bicarbonate ratio of 1:5. Even though the systems are identical in a global sense, the measured pH value is different between the systems which would lead to incorrect interpretations if the two-phase system is not considered.

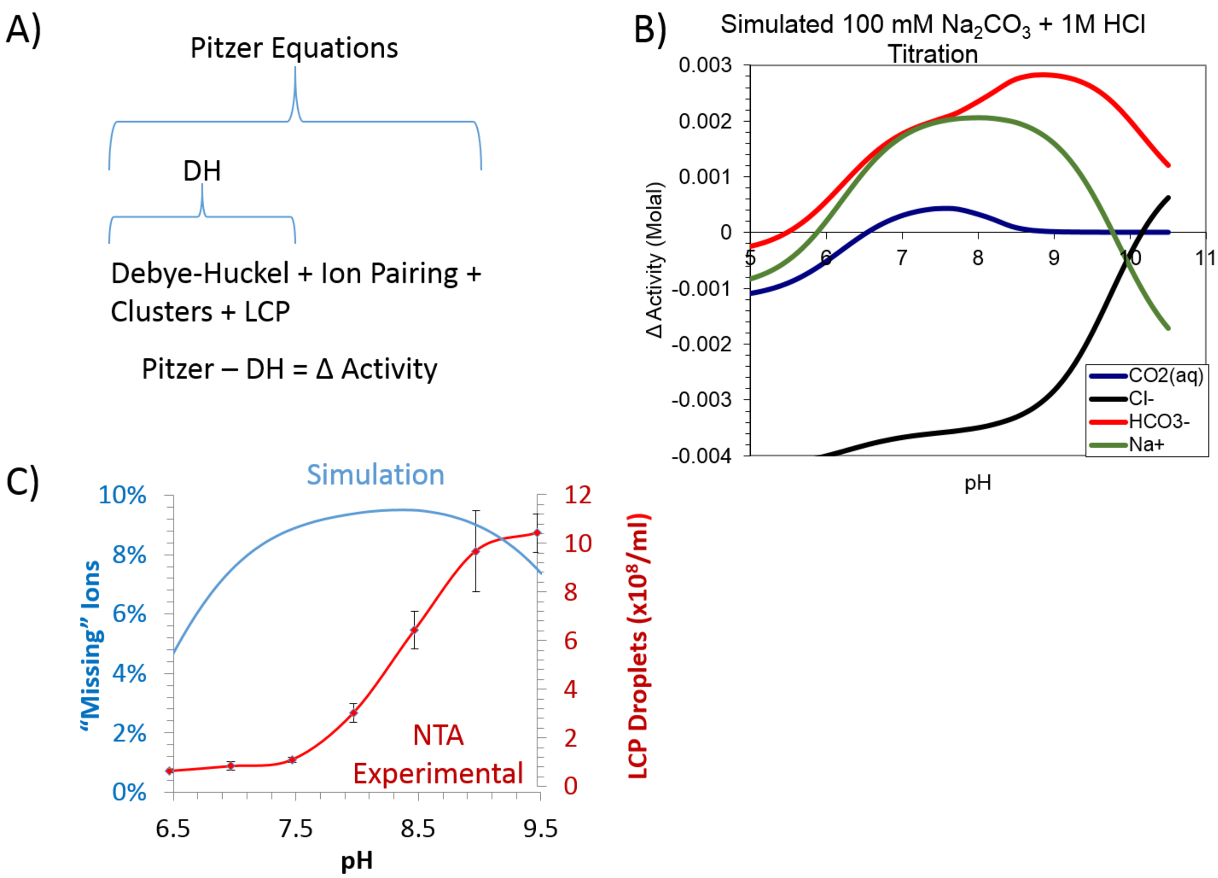

Fig. S7. The concentration of bicarbonate-rich liquid LCP droplets (as measured by nanoparticle tracking analysis) qualitatively matches the activity drop of ions predicted from our thermodynamic analysis. (**A**) Pitzer equations model bicarbonate solutions empirically which considers all of the known contributions to activity loss. Debye- Hückel, models only consider columbic interactions. By subtracting the activities predicted by Pizter Equations from those predicted by D-H theory, we isolate the activity loss of ions due to bicarbonate-rich LCP. Ion pairing and prenucleation clustering is negligible due to the neutral pH, lack of divalent ions, and the weak ion associations known for all three ions (Na^+, Cl^-, HCO_3^-). (**B**) The difference in ion activity between actual ion activities (approximated using Pitzer's equations) and that predicted by Debye-Hückel theory for a 50 mM $NaHCO_3$ with varying amounts of HCl added to adjust pH. Species with positive deviations (lower activities) are interpreted as strong candidates to be participating in the LCP phase. Negative deviations are interpreted to be species that are relatively enriched in the bulk phase and are excluded from the LCP. The composition of the LCP phase is then the sum of the positive deviations of the curves, which provides the amounts of species missing from bulk solution (**C**) The comparison of measured LCP droplet concentrations via NTA vs. the predicted percent of ions participating in LCP as predicted from part B. The NTA experimental data was collected in triplicate and is presented as an average with a standard deviation of error in either direction. The amount of LCP droplets detected by NTA decreases in a way qualitatively similar to our prediction. The drop in concentration of LCP droplets detected occurs more rapidly than the drop according to the Pitzer equations. This suggests that the LCP droplets may be smaller as well as fewer as the pH is lowered and therefore are not being detected by the NTA (which has a size detection lower limit of 40 nm). We speculate that there may be a spinodal for the LCP below pH 7.5. This would be consistent with an LCP phase that is bicarbonate-rich and slightly acidic as reported previously as the energy of interfacial formation would be reduced in this environment.

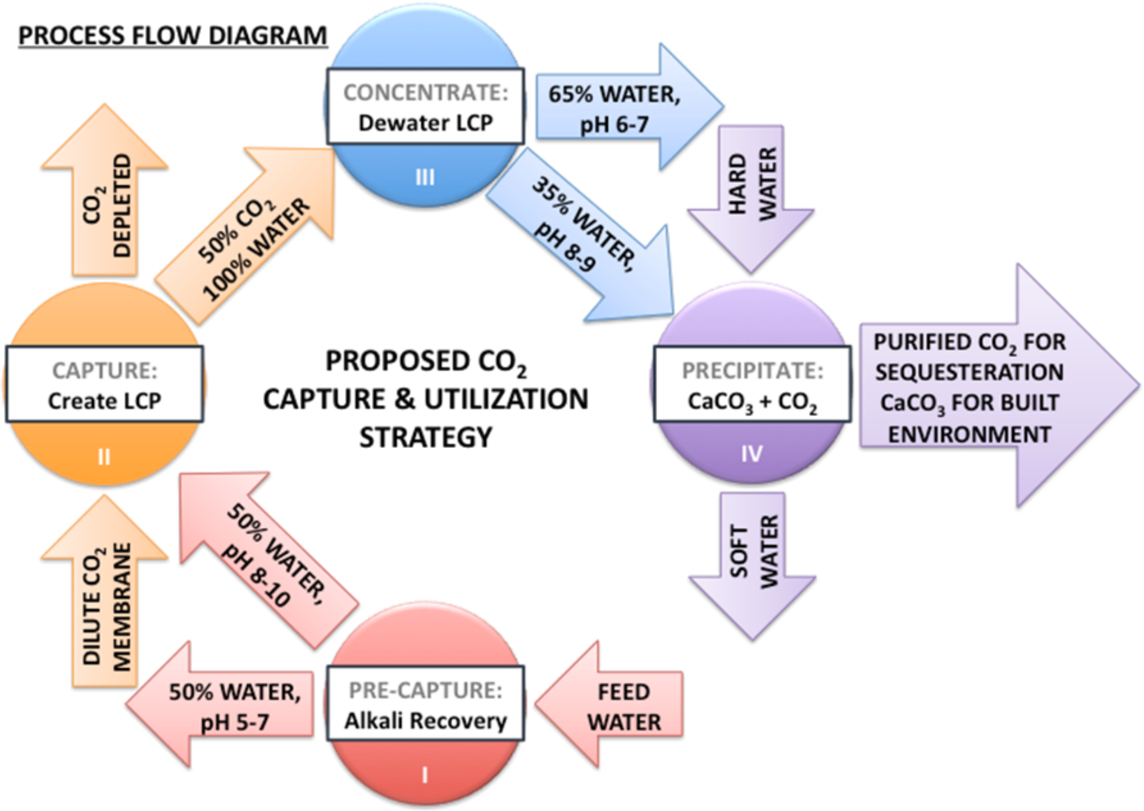

Fig. S8. A flow process diagram illustrating the proposed carbon sequestration mechanism allowed by the discovery of bicarbonate-rich LCP. The four stages of the process are: (I) Pre-Capture: the creation of the CO_2 capture solution, (II) Capture: the capture solution is contacted with flue gas, (III) Concentrate: dilute solutions of LCP are concentrated by membrane dewatering, and (IV) Precipitate: a hard water source is combined with the concentrated LCP solution to precipitate synthetic limestone (calcium carbonate, $CaCO_3$).

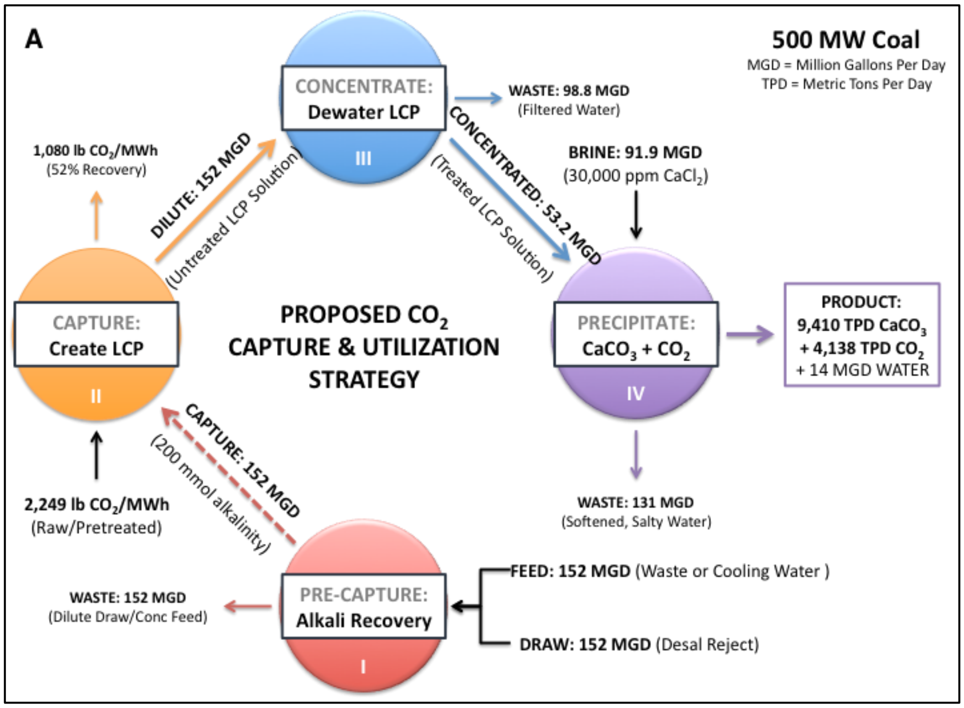

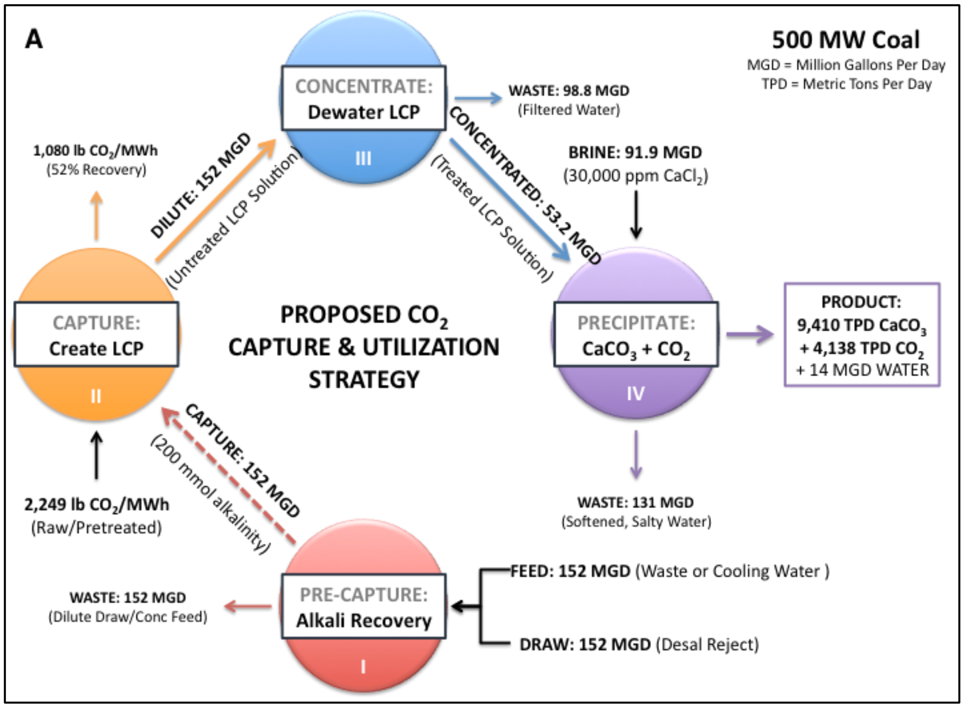

Fig. S9. Models for the LCP Technology in place at a 500 MW power plant. (**A**) Coal-fired power plant. (**B**) Natural gas-fired power plant. Each case assumes the solution developed at the Pre-Capture stage will have 200 mmol alkalinity available to capture CO_2, and that the $CaCO_3$ precipitation occurs by reaction of one equivalent of Ca^{2+} with two equivalents of HCO_3^-.

References and Notes:

1. V. Filipe, A. Hawe, W. Jiskoot, Critical Evaluation of Nanoparticle Tracking Analysis (NTA) by NanoSight for the Measurement of Nanoparticles and Protein Aggregates. *Pharmaceutical Research* **27**, 796 (2010).
2. C. Gardiner, Y. J. Ferreira, R. A. Dragovic, C. W. G. Redman, I. L. Sargent, Extracellular vesicle sizing and enumeration by nanoparticle tracking analysis. *Journal of Extracellular Vesicles* **2**, 19671 (2013).
3. E. van der Pol *et al.*, in *International Society for Extracellular Vesicles 2014*. (Rotterdam, Netherlands, 2014).
4. *ASTM Standard C109/C109M, Standard Test Method for Compressive Strength of Hydraulic Cement Mortars (Using 2-in. or [50-mm] Cube Specimens).* (ASTM International, West Conshohocken, PA, 2013).
5. *ASTM Standard C305, Standard Practice for Mechanical Mixing of Hydraulic Cement Pastes and Mortars of Plastic Consistency*. (ASTM International, West Conshohocken, PA, 2013).
6. R. Levinson, H. Akbari, P. Berdahl, Measuring solar reflectance - Part I: Defining a metric that accurately predicts solar heat gain. *Solar Energy* **84**, 1717 (2010).
7. R. Levinson, H. Akbari, P. Berdahl, Measuring solar reflectance - Part II: Review of practical methods. *Solar Energy* **84**, 1745 (2010).
8. *ASTM Standard E892-87 for Terrestrial Solar Spectral Irradiance at Air Mass 1.5 for a 37-Deg tilted surface*. (ASTM International, West Conshohocken, PA, 1992).
9. *National Ready-Mix Concrete Association, Concrete CO2 Fact Sheet, based on the most recent survey of Portland Cement Association members*. (Feb 2012).
10. I. Zondervan, R. E. Zeebe, B. Rost, U. Riebesell, Decreasing marine biogenic calcification: A negative feedback on rising atmospheric pCO2. . *Global Biogeochemical Cycles* **15**, 507 (2001).
11. K. Naka, Y. Tanaka, Y. Chujo, Effect of Anionic Starburst Dendrimers on the Crystallization of CaCO3 in Aqueous Solutinon: Size Control of Spherical Vaterite Particles. *Langmuir* **18**, 3655 (2002).
12. L. Addadi, S. Raz, S. Weiner, Taking advantage of disorder: amorphous calcium carbonate and its roles in biomineralization. *Adv. Mater.* **15**, 959 (2003).
13. J. D. Rodriguez-Blanco, S. Shaw, L. G. Benning, The kinetics and mechanisms of amorphous calcium carbonate (ACC) crystallization to calcite, via vaterite. *Nanoscale* **3**, 265 (2011).
14. E. Loste, R. M. Wilson, R. Seshadri, F. C. Meldrum, The role of magnesium in stabilising amorphous calcium carbonate and controlling calcite morphologies. *Journal of Crystal Growth* **254**, 206 (2003).
